# Superior Vena Cava Syndrome. From the Bronchus to the Vessel

**DOI:** 10.1155/DTE.4.83

**Published:** 1997

**Authors:** C. Witt, B. Schmidt, A. C. Borges, W. Doerffel, G. Baumann, P. Romaniuk

**Affiliations:** 1 Division of Pneumology Department of Internal Medicine I Medical School (Charité) Humboldt-University Berlin Germany; 2 Division of Interventional Radiology Department of Radiology Medical School (Charité) Humboldt-University Berlin Germany

## Abstract

This paper addresses the diagnosis and management of superior vena cava syndrome (SVCS) due to malignant intrathoracic tumours. Diagnosis of SVCS is usually established by bedside examination. Chest X-ray and computed tomography may be helpful, but the cavography remains the “gold-standard”. Other imaging techniques (MRI, nuclear flow studies) are more of scientific interest. Bronchoscopy helps to evaluate the risk of pulmonary complications and endoscopic procedures often lead to histological findings. In the treatment of malignant SVCS surgery, radiotherapy, and chemotherapy have been successfully used. The placement of a vascular stent might be an additional or alternative possibility. There are no conclusive indication criteria and no conclusive regimen concerning post-stenting anticoagulation. From all reported results and published papers we draw the conclusion that the immediate effects of stent implantation and the long-term results of tumour-specific therapy are complementary to one another. The stent dilates the local venous stenosis while tumour-specific therapy has a general effect on the vascular and respiratory situation in a multi-therapy concept.


*Diagnostic and Therapeutic Endoscopy*, Vol. 4, pp. 83–93
Reprints available directly from the publisher
Photocopying permitted by license only

© 1997 OPA (Overseas Publishers Association)
Amsterdam B.V. Published under license
in The Netherlands under the Harwood Academic
Publishers imprint, part of The Gordon and
Breach Publishing Group.
Printed in Singapore.

### Superior Vena Cava Syndrome. From the Bronchus to the Vessel – A Review

C. WITT[a,*], B. SCHMIDT[a], A.C. BORGES[a], W. DOERFFEL[a], G. BAUMANN[a] and P. ROMANIUK[b]

[a] *Division of Pneumology, Department of Internal Medicine I,* [b] *Division of Interventional Radiology, Department of Radiology, Medical School (Charité), Humboldt-University, Berlin, Germany*

*(Received 26 December 1996; Revised 10 March 1997; In final form 8 May 1997)*

**This paper addresses the diagnosis and management of superior vena cava syndrome (SVCS) due to malignant intrathoracic tumours. Diagnosis of SVCS is usually established by bedside examination. Chest X-ray and computed tomography may be helpful, but the cavography remains the "gold-standard". Other imaging techniques (MRI, nuclear flow studies) are more of scientific interest. Bronchoscopy helps to evaluate the risk of pulmonary complications and endoscopic procedures often lead to histological findings. In the treatment of malignant SVCS surgery, radiotherapy, and chemotherapy have been successfully used. The placement of a vascular stent might be an additional or alternative possibility. There are no conclusive indication criteria and no conclusive regimen concerning post-stenting anticoagulation. From all reported results and published papers we draw the conclusion that the immediate effects of stent implantation and the long-term results of tumour-specific therapy are complementary to one another. The stent dilates the local venous stenosis while tumour-specific therapy has a general effect on the vascular and respiratory situation in a multi-therapy concept.**

*Keywords:* Superior vena cava syndrome, Stent implantation, Bronchoscopy

### SYMPTOMS AND CAUSES OF SUPERIOR VENA CAVA SYNDROME

The superior vena cava (SVC) is the the major drainage system for blood from the upper part of the body including the head and the arms. It is a thin walled vessel measuring ca. 6 cm in length and 1.5–2.0 cm in width, and is formed by the union of both brachiocephalic veins and leading towards the right atrium. The azygos vein is the only vein that enters the SVC, it carries blood from the thoracic wall and the thoracic organs.

The superior vena cava syndrome (SVCS) is characterized by venous congestion of the upper extremities, head, and neck in most of the patients [1] (Fig. 1). While these symptoms often appear slowly and are not noticed by the patient severe dyspnoea often makes the patient look for medical advice. The dyspnoea is usually aggravated by bending forward and in supine position [1], which

* Corresponding author. Tel.: 49-30-2802 4012. Fax: 49-30-2802 8691.

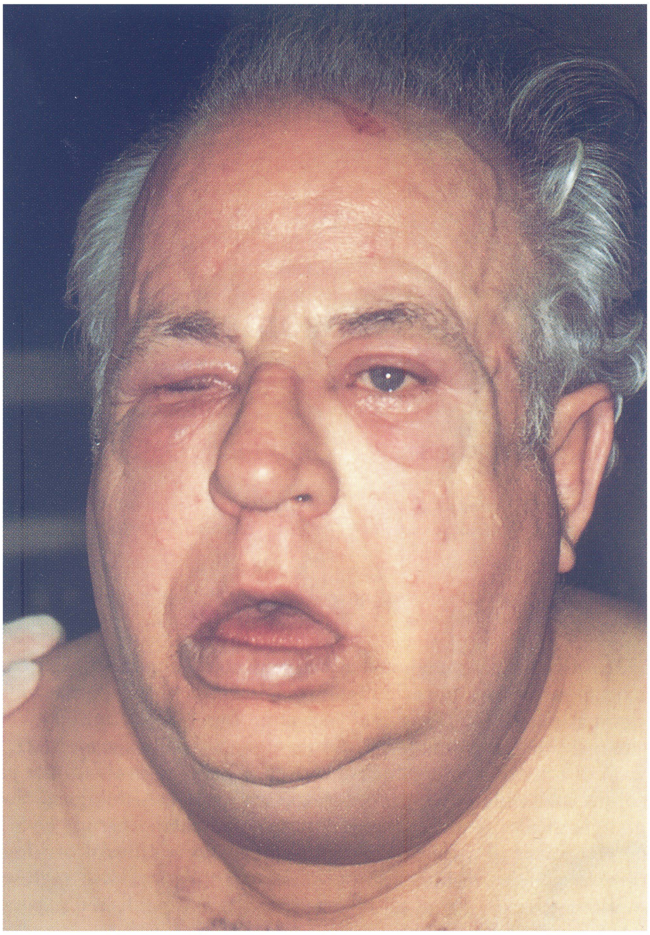

FIGURE 1 Swelling of head and neck due to severe venous congestion in a patient with SVCS due to bronchial carcinoma.

makes the patient looking for an upright position at night. Oedema of the glottis is a life-threatening effect of the venous congestion [2]. Collateral veins at the thoracic wall are visible in the majority of cases if the condition has existed for any length of time [3]. Cyanosis is present in 13–20% of the patients [1,4], neurological symptoms like headache, alteration in the state of consciousness, and visual disturbance are found in varying frequencies [1,5–8].

*Causes:* In 85–97% SVCS occurs due to malignant intrathoracic tumors [9]. In a large review of 125 cases by Armstrong *et al.* pulmonary tumours were responsible for 80% of the SVCS, further 14% were due to thoracic lymphomas [4]. Other tumours like leiomyosarcoma of thoracic vessels or plasmocytoma have been reported to cause SVCS in single cases [10,11]. On the other hand in 5–15% of cases with intra-thoracic malignant tumours SVCS developes [12,13].

Benign causes include tuberculous mediastinitis, syphilitic aortic aneurysms, mediastinal fibrosis, post-irradiation fibrosis, posttraumatic strictures, and intrathoracic goiter [14–18]. Iatrogenic causes for SVCS like catheter manipulations, pacemaker wire implantation, and aortic valve replacement must also be taken into consideration [19–21].

This article addresses the influence of intra-thoracic tumours on the vena cava superior and the tracheobronchial system. We want to give an overview about the clinical value of vascular and bronchological diagnostics and therapeutics in SVCS due to intrathoracic tumours.

### VASCULAR, IMAGING AND BRONCHOLOGICAL DIAGNOSTICS

Diagnosis of the SVCS is established by bedside examination of the patient in a high percentage of patients. Further diagnostic procedures include chest X-ray, computed tomography (CT), venography, radionuclide studies, Doppler flow studies, magnetic resonance imaging (MRI), and bronchoscopy.

*Chest X-ray* proves useful in 67–78% showing a superior mediastinal enlargement, a right hilar mass, and the intra-thoracic tumour [1,4]. These changings are not specific and require further investigations. *CT* provides more accurate information on localization of the obstruction and size of the tumour [14]. It also shows whether the SVC is totally or partially surrounded by tumour or whether the vessel is tangential to the tumour. These details may predict the outcome after stent implantation [22]. The *venography* (Fig. 2) is an invasive procedure and is usually performed simultaneously via both cubital veins [1,5,18,23,24]. Its disadvantages are the use of iodinated contrast media with the inherent risk of anaphylactic reaction and the increased risk of thrombus formation [14]. Nevertheless, the cavography remains the "gold-standard" in the detection of venous stenosis and occlusion (Fig. 2). It is also essential to

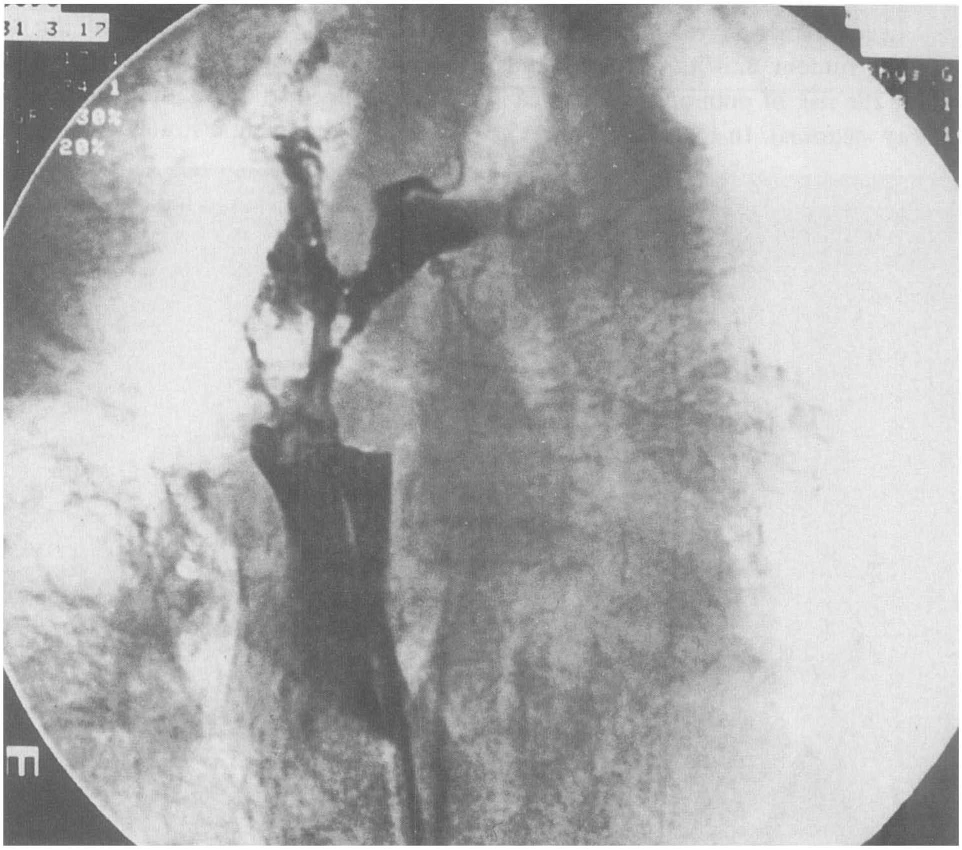

FIGURE 2 Venogram of a patient with severe stenosis of the superior vena cava.

establish the pattern of venous collaterals prior to surgical placement of bypass grafts [25] (Fig. 3). *Nuclear flow* studies with 99 m Technetium labeled microspheres which are less thrombogenic than iodinated contrast media are not routinely performed as their results are less accurate [1,14]. *Doppler flow studies* are useful for investigations of peripheral venous flow (axillary, subclvian, and jugular veins) and venous congestion in SVCS but are of limited use in intra-thoracic veins [26,27]. *MRI* seems valuable in diagnosing intra-thoracic venous obstruction with a sensitivity of 94% and specificity of 100%, but seems less suitable for evaluating patency after endoprothesis placement because of stent-induced artefacts [28]. It is not routinely performed.

Prospective studies about the role of *bronchoscopy* in SVCS do not exist. However, endocopic techniques (inspection, broncho-alveolar lavage, forceps biopsy, needle biopsy) give valuable informations about the tumour and its histology and helps to evaluate the risk of pulmonary complications (e.g. airway stenoses). In the first of our 13 patients who presented with SVCS and who received a venous stent we observed tracheal compression in 11 cases, right main or intermediate bronchi compression in eight cases, and stenosis of the left main bronchus in two cases (Table I). Only one patient did not show any abnormal bronchoscopical picture. In most cases bronchoscopic procedures (biopsy, needle aspiration, transbronchial biopsy) lead to reliable histolgical findings. Therapeutic bronchoscopy is necessary in certain patients with compression of the SVC and central airways simultaneously or in the follow up. In this case stenting of the airway and SVC may be indicated (Figs. 4(a), (b)).

### TREATMENT OF SVCS

Symptomatic improvement is often achieved by oxygen, analgesia, elevation of the head of the bed, diuretics, and corticosteroids; thereby time can be gained to assess the situation and develop a suitable therapeutic strategy [14].

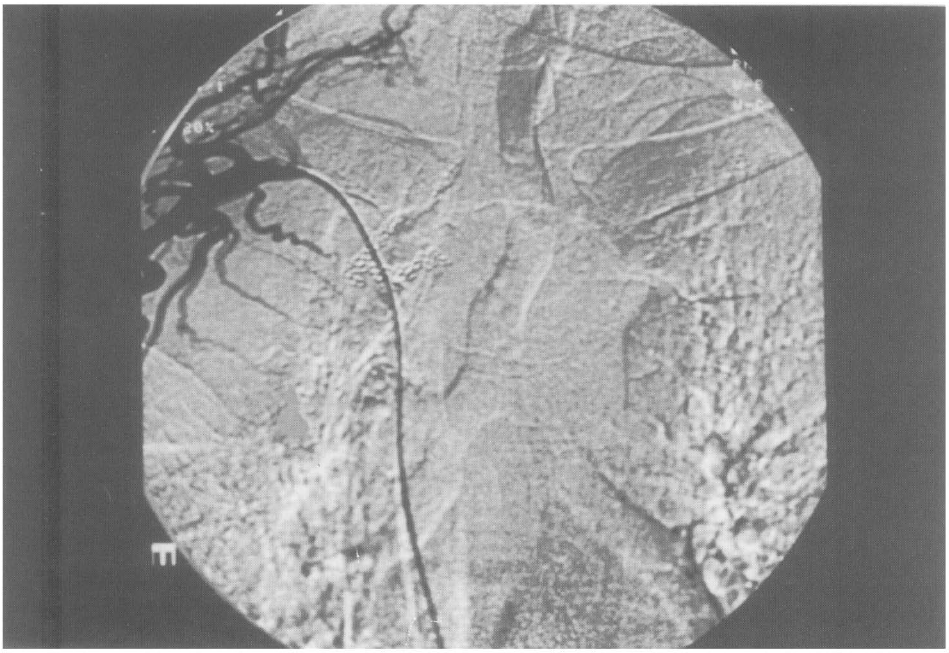

FIGURE 3 Venous collaterals in a patient with severe SVCS.

Surgery, radiotherapy, and chemotherapy have been used to treat SVCS due to malignant obstruction [12,29–33]. Surgical bypass of the SVC requires thoracotomy which is best avoided in most patients with end-stage malignant disease [31,34], nevertheless there are several papers describing successful palliation achieved by surgical intervention [25,35].

Radiotherapy is a well established treatment modality for malignant SVCS but the response

TABLE I Findings in the first 13 patients with SVCS treated with a venous stent. (No. = number, SCLC = small cell lung carcinoma, NSCLC = non-small cell lung carcinoma, SVC = superior vena cava, jugular = right jugular vein, brachioceph = brachiocephalic trunc)

| No. | Age/sex | Tumour | Airway stenoses | | | Venous stenoses | | |
|---|---|---|---|---|---|---|---|---|
| 1 | 62/f | NSCLC | trachea | — | left | SVC | jugular | — |
| 2 | 68/m | SCLC | trachea | right | — | SVC | — | brachioceph |
| 3 | 47/m | M. Hodgkin | — | — | — | SVC | — | — |
| 4 | 52/m | NSCLC | trachea | right | — | SVC | jugular | brachioceph |
| 5 | 73/f | thyroid ca | trachea | — | left | SVC | — | — |
| 6 | 77/m | SCLC | — | right | — | SVC | — | brachioceph |
| 7 | 64/m | SCLC | trachea | right | — | SVC | — | brachioceph |
| 8 | 66/m | NSCLC | trachea | — | — | SVC | — | brachioceph |
| 9 | 76/m | SCLC | trachea | right | — | SVC | — | brachioceph |
| 10 | 58/m | NSCLC | trachea | right | — | SVC | — | — |
| 11 | 50/m | SCLC | — | right | — | SVC | jugular | — |
| 12 | 72/f | NSCLC | trachea | — | — | SVC | jugular | — |
| 13 | 63/f | metastasis | trachea | right | — | SVC | — | — |

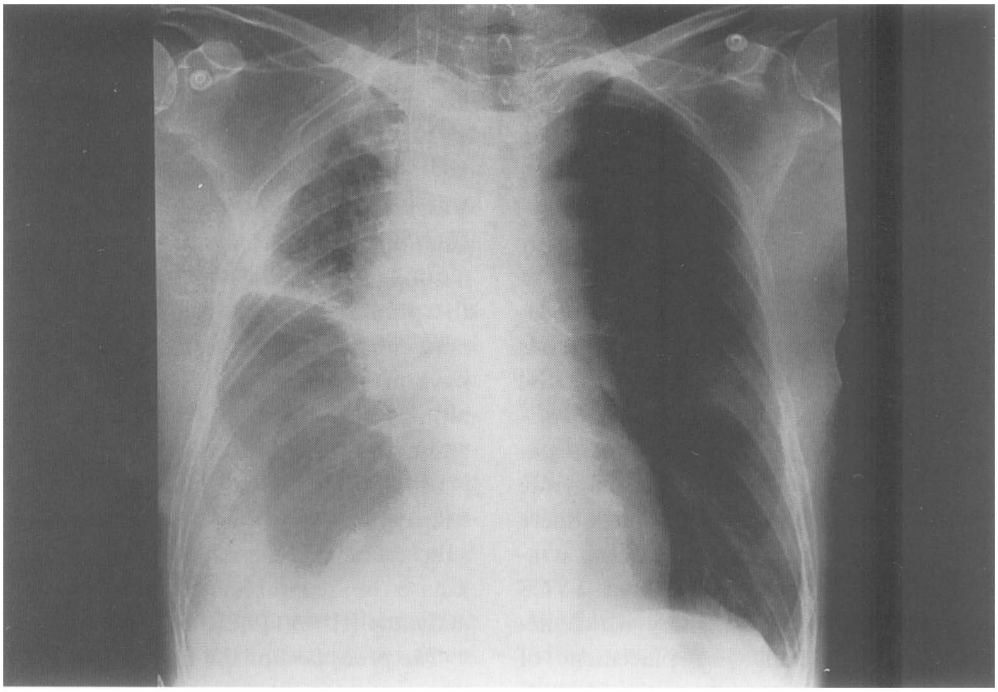

FIGURE 4(a) In some patients vascular and airway stents have to be implanted.

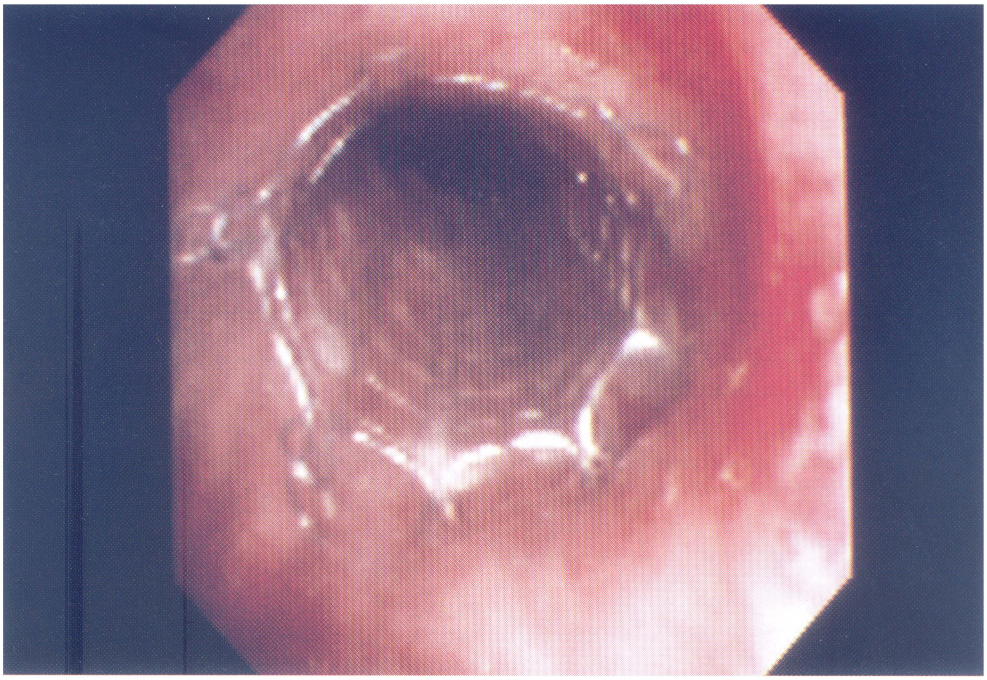

FIGURE 4(b) Endobronchial Strecker-stent in a patient with malignant SVCS (Figs. 4(a), (b) taken from the same patient).

may be delayed for 2–3 weeks [34]. Armstrong *et al.* report complete symptomatic relief in the majority of patients with lymphoma and partial relief in ca. 50% of patients with bronchogenic carcinoma [4]. Irving *et al.* achieved relief in 90% of the patients with malignant stenoses within three weeks [17].

In small-cell lung cancer (SCLC) chemotherapy is often effective. Sculier *et al.* report success of chemotherapy in terms of symptomatic improvement in 73% of the patients [32]. In another series of 37 patients with SCLC complete or partial relief was demonstrated in 84% [33].

However all tumour-specific treatment strategies require some time to show effect, and some tumours do not respond at all. Furthermore there might be tumour recurrence after maximum irradiation or extensive chemotherapy and SVCS sometimes re-occurs as an emergency which necessitates immediate action. The placement of vascular stents seems to be an alternative in these situations. There are numerous reports about the use of Gianturco-, Wall-, and Palmaz-stents in SVCS (Table II).

### VENA CAVA STENTING

Various stent types have been developed for vascular and extra-vascular indications (biliary stents, oesophageal stents, tracheobronchial stents). In the published papers about SVCS due to malignant conditions Gianturco-Z-stents and Wallstents have mostly been used [36–40]. There are several reports about Palmaz balloon-expandable intravascular stents in benign and malignant SVCS [41–44]. Prior to stent placement thrombolysis has been performed in some patients with SVCS or subclavian thrombus using Streptokinase, Urokinase or recombinant tissue-type plasminogen activator (rt-PA) [6,23,45–47].

*The procedure* of the implantation is performed via percutaneous femoral [5,7,8,18,23,48,49–51], antecubital [18,51] or jugular [7,22] vein approach,

TABLE II Published studies, venous stent implantation in malignant superior vena cava syndrome. (w = Wallstent, g = Gianturco-Z-stent, rec = recurrence in *n* cases, d = days, × = no information)

| Author | Year | Number of patients | Immediate relief (%) | rec | Time until rec |
|---|---|---|---|---|---|
| Antonucci | 1992 | 2 w | 100 | 1 | 90 d |
| Crowe *et al.* | 1995 | 11 g | 100 | 5 | 14–183 d, thrombosis |
| Dyet *et al.* | 1993 | 17 w | 100 | 2 | 120 d, thrombosis |
| Edwards | 1993 | 1 g | 100 | 0 | 90 d |
| Elson *et al.* | 1991 | 5 p | 100 | 0 | × |
| Ferro *et al.* | 1995 | 9 g | 100 | 2 | × |
| Furui *et al.* | 1995 | 16 g | 81 | 2 | 21 d |
| Gaines *et al.* | 1994 | 20 g | 90 | 3 | × |
| Hennequin *et al.* | 1995 | 14 w | 100 | 1 | 90 d, thrombosis |
| Irving *et al.* | 1992 | 17 g | 94 | × | × |
| Kishi *et al.* | 1993 | 6 g | 100 | 1 | 60 d, local metastasis |
| Köhler *et al.* | 1995 | 1 w | 100 | 1 | 130 d, thrombosis |
| Link *et al.* | 1995 | 4 w | 100 | 0 | 2 d |
| Oudkerk *et al.* | 1992 | 14g + w | 71 | 3 | 21 d |
| Putnam *et al.* | 1988 | 2 g | 100 | 0 | 30 d |
| Rösch *et al.* | 1992 | 20 g | 100 | 1 | 270 d, tumour ingrowth |
| Sawada *et al.* | 1991 | 6 g | 100 | 1 | 49 d |
| Solomon *et al.* | 1991 | 6 g | 100 | 2 | 28 d |
| Stock *et al.* | 1995 | 14 w | 85 | 3 | 7/78/247 d, thrombosis |
| Tacke *et al.* | 1994 | 8 g + w | 100 | 0 | 60 d, tumour compression |
| Watkinson *et al.* | 1993 | 4 w | 100 | × | × |
| Wilhelm *et al.* | 1995 | 14 g + w | 85 | 0 | 60 d |

depending on the location of the SVC-stricture and the presence of additional occlusions in the brachiocephalic veins. Prior to insertion of the stent balloon dilatation can be performed [22,50], it may be useful especially in those patients who have short, and very tight stenoses [34]. The dilatation balloon also serves to delineate the extent of the stricture [7]. The introducer catheter is then advanced and the stent positioned across the stenotic area. The stent is deployed by carefully withdrawing the sheath. If more than one stent is used the most distal one is placed first followed by the more proximal ones in an overlapping arrangement [7]. Once the stent is in place a dilatation balloon inside the stent may be used to accelerate it's expansion and dilatate any remaining stricture [5,7,18,23]. During the implantation procedure a bolus injection of 5000 IU heparin (iv) is given by most authors [18,23,51,52].

In the *post-interventional treatment* there is no concurrent strategy for post-stenting treatment concerning dosages and applications of antithrombotic and thrombolytic drugs. Thrombolytic drugs were used by Kishi *et al.*, Furui *et al.*, and Sawada *et al.* [5,22,50] to prevent thrombotic re-occlusion in patients who had severe thrombosis of the SVC prior to stenting or where thrombi were seen at post interventional cavography. Oral anticoagulation was performed during 3 or 6 months following stent implantation in patients with pre-stenting thrombosis [18,23]. Others did not perform oral anticoagulation at all, even if thrombotic occlusion was present prior to stent implantation [52]. And Rösch *et al.* advocate lifelong anticoagulation after stenting in all patients with malignant SVCS [7]. Heparin was given at therapeutic dosage for 1–5 days after the procedure in most of the published series [7,8,22,23, 45,51–53]. Anti-platelet therapy was performed by Kishi *et al.* using 1000 mg ASS (iv) for 7 days and 3000 mg (po) for another 7 days [5]; Dyet *et al.* used 300 mg ASS for 3 months [23], and Watkinson gave 75 mg until patients death [8]. Most other papers do not report any anti-platelet therapy as its effect in the venous system seems to be uncertain [51,54].

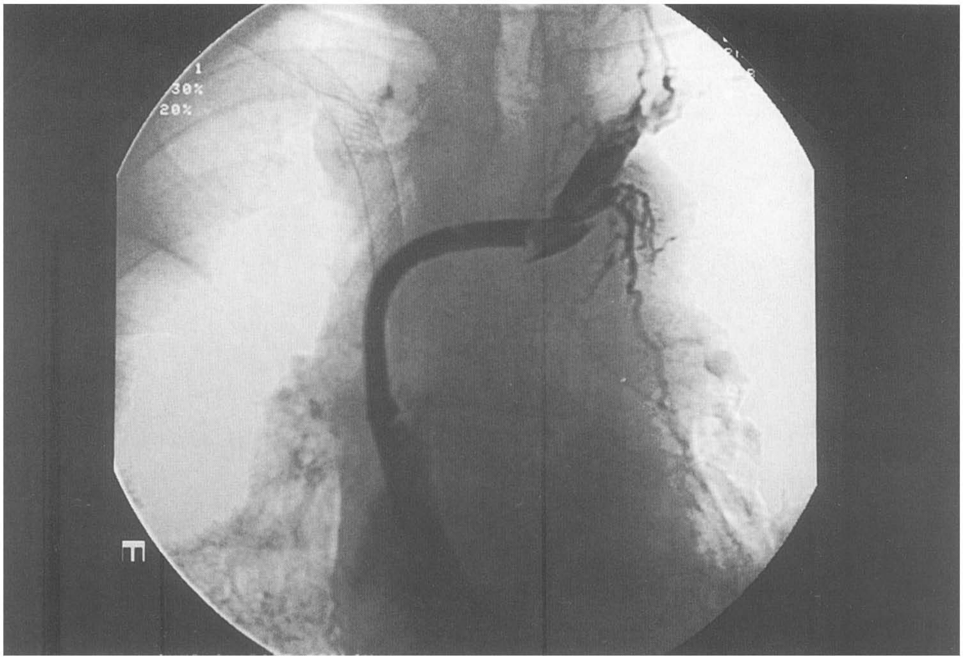

FIGURE 5 Free passage of contrast media through the stented vessel (left brachiocephalic vein and SVC).

*Stent insertion* leads to a clinical improvement of the venous congestion in 71% [24] to 100%, analyzing the present papers the overall success rate is estimated to be over 90% (Table II). Clinical features of SVCS are reported to decrease markedly within 48 h after the procedure [6,18,23] (Fig. 5).

Stent insertion was not successful when the implantation catheter could not be safely advanced through the stenostic area [18], when strictures were to tight for dilatation [22], when the anatomic situation made stent positioning difficult [51], and when Gianturco-stents broke after the implantation [24]. Immediate *complications* of the stenting procedure rarely appeared. Pulmonary oedema due to sudden overload of the heart when venous return normalizes is one of them and needs immediate treatment [5]. Short-term complications of SVC stent implantation are thrombosis, SVCS-recurrence, and technical complications.

Thrombotic occlusion of the stent seems to be a common complication and may occur in spite of heparin treatment [52]. When anticoagulation is discontinued, e.g. after appearance of severe side-effects [23], thrombosis of the stented SVC may appear [18].

Recurrence of SVCS due to tumour progression has been reported by several authors, it may cause compression of the stented SVC [55] or may lead to ingrowth of tumorous tissue into the lumen of the vessel [7].

Oudkerk *et al.* report three cases of Gianturco-stent fracture with a migrating fragment in one case [24], another technical complication, Gianturco-stent migration, is reported by Furui *et al.* [22].

### DISCUSSION

*Indications* for stent implantation in malignant SVCS have not been well defined. Care has to be taken as the stent once implanted and completely released cannot be removed by other means than surgical intervention. In most series stents were implanted in patients who already underwent radiotherapy or chemotherapy and do not respond sufficiently, in patients with recurrent SVCS, and in patients who refused tumour-specific therapy [22–24,56]. Kishi *et al.* defined rather strict indication criteria which included elevated caval pressure (> 22 mmHg), absence of vascular compensation, and absence of other anticancer therapy, furthermore patients cardiac function had to be good enough to tolerate possible overload [5]. Most suitable lesions for stent implantation are stenoses with not completely obstructed lumina which allow free passage of the guide wire and the introductory sheath [7].

Most authors felt that intraluminal tumour or thrombus formation should be a *contraindication* to stent placement [5,8,23]. Oudkerk *et al.* applied the following contraindications: chronic complete vessel occlusion proved by imaging, severe coagulopathy, and chronic cardiac disease [24].

In the end there is little doubt that stent implantation is the procedure of choice in patients with recurrent SVCS who already underwent maximum radio- or chemotherapy.

Whether stent implantation in the SVC should be performed as first line treatment remains

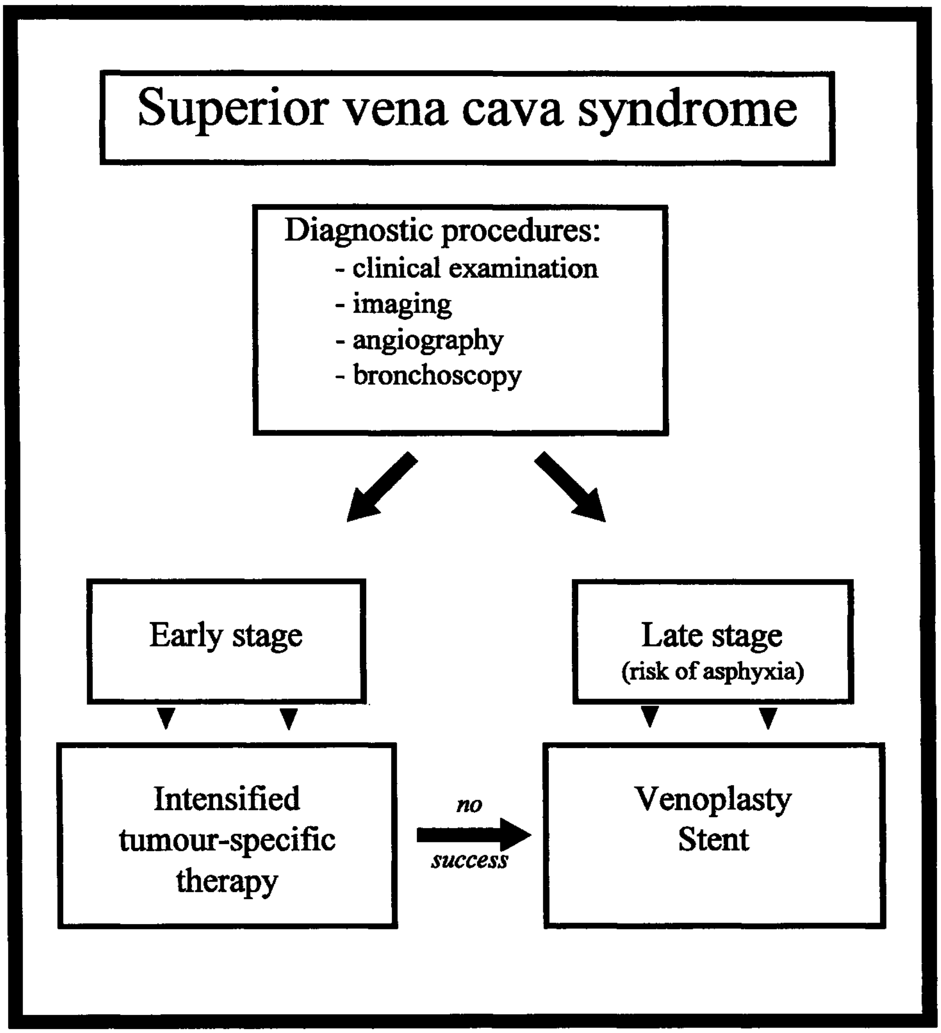

FIGURE 6 Strategy in the treatment of the superior vena cava syndrome.

controversial. On the one hand some statistical findings indicate, that in 80% of SVCS due to small-call lung cancer chemotherapy successfully reduces venous congestion [33,57]; in non-small cell lung cancer response rates of radiotherapy are reported to be between 65% and 90% within three weeks [4,17]. On the other hand stent implantation is an easy and relatively safe procedure in the hand of an experienced specialist, it gives immediate relief of SVCS symptoms, which is important in patients with limited life expectancy; furthermore it does not interfere at all with any subsequent therapy [34]. For Dyet *et al.* [23] stenting without prior tumour-specific therapy is justified in patients with very severe symptoms who would not tolerate or survive radio- or chemotherapy.

From all reported results and published papers we draw the conclusion, that stent implantation and tumour-specific therapy should be combined. The stent dilates the local venous stenosis while tumour-specific therapy has a general effect on the vascular and respiratory situation. In the early stage radio- or chemotherapy should be intensified, in late stage SVCS stent implantation should be performed as first line treatment (Fig. 6).

### *References*

[1] Kretschmer, S. and W. Schneider, Obere Einflußstuung als onkologischer Notfall. *DMW* 1992; **117**: 1650–1655.

[2] Köhler, F., P. Romaniuk, C. Witt, D. Stockheim, H.G. Mergenthaler, R. Grunewald and G. Baumann, Venöse Angioplastie und Wallstent-Implantation in der Notfalltherapie eines tumorbedingten Vena-cava-superior-Syndroms. *DMW* 1995; **120**: 1074–1079.

[3] Okay, N.H. and D. Bryk, Collateral pathways in occlusion of the superior vena cava and its tributaries. *Radiology* 1969; **92**: 1493–1498.

[4] Armstrong, B.A., C.A. Perez and J.R. Simpson, Role of irradiation in the management of superior vena cava syndrome. *Int. J. Radiat. Oncol. Biol. Phys.* 1987; **13**: 1238–1242.

[5] Kishi, K., T. Sonomura, K. Mitsuzane, N. Nishida, R. Yang and M. Sato, Self-expandable metallic stent therapy for superior vena cava syndrome: clinical observations. *Radiology* 1993; **189**: 531–535.

[6] Putnam, J.S., B.T. Uchida, R. Antonovic and J. Rösch, Superior vena cava syndrome associated with massive thrombosis: treatment with expandable wire stents. *Radiology* 1988; **167**: 727–728.

[7] Rösch, J., B.T. Uchida, L.D. Hall, R. Antonovic, B.D. Petersen, K. Ivancev, R.E. Barton and F.S. Keller, Gianturco-Rösch expandable Z-stents: clinical experience in the vena cava and large veins. *Cardiovasc. Intervent. Radiol.* 1992; **15**: 328–333.

[8] Watkinson, A.F. and D.M. Hansell, Expandable Wallstent for the treatment of obstruction of the superior vena cava. *Thorax* 1993; **48**: 915–920.

[9] Perez, C.A., C.A. Presant and A.L. van Amburg, Management of superior vena cava syndrome. *Semin. Oncol.* 1978; **5**: 123–134.

[10] Davis, S.R., H.S. King and R.I. Le, Superior vena cava syndrome caused by an intrathoracic plasmocytoma. *Cancer* 1991; **68**: 1376–1379.

[11] Weiss, K.S., B.L. Zidar and S. Wang, Radiation-induced leiomyosarcoma of the great vessels presenting as superior vena cava syndrome. *Cancer* 1987; **60**: 1238–1242.

[12] Davenport, D., C. Ferree, D. Blake and M. Raben, Radiation therapy in the treatment of superior vena caval obstruction. *Cancer* 1978; **42**: 2600–2603.

[13] Dyet, J.F. and K. Moghissi, Role of Venography in assessing patients with superior vena caval obstruction caused by carcinoma for bypass operations. *Thorax* 1980; **8**: 628–630.

[14] Abner, A., Approach to the Patient who Presents with Superior Vena Cava Obstruction. *Chest* 1993; **103**: 394S–397S.

[15] Cengiz, K., A. Aykin and A. Demirci, Intrathoracic goiter with hypothyroidism, tracheal compression, superior vena cava syndrome, and Horner's syndrome. *Chest* 1990; **97**: 1005–1006.

[16] Hennequin, L.M., O. Fade, J.G. Fays, J.F. Bic, S. Jaafar, A. Bertal, D. Anthoine and P.A. Bernadac, Superior vena cava stent placement: results with the Wallstent endoprothesis. *Radiology* 1995; **196**: 353–361.

[17] Irving, J.D., R.F. Dondelinger, J.F. Reidy, H. Schild, R. Dick, A. Adam, M. Maynar and C.L. Zollikofer, Gianturco self-expanding stents: clinical experience in the vena cava and large veins. *Cardiovasc. Intervent. Radiol.* 1992; **15**: 328–333.

[18] Stock, K.W, A.L. Jacob, M. Proske, C.T. Bolliger, C. Rochlitz and W. Steinbrich, Treatment of Malignant Obstruction of the Superior Vena Cava with the Self-expanding Wallstent. *Thorax* 1995; **50**: 1151–1156.

[19] Aebischer, N., A.J. Shurman and S. Sharma, Late localized tamponade causing superior vena cava syndrome: an unusual complication of aortic valve replacement. *Am. Heart. J.* 1988; **115**: 1130–1132.

[20] Bertrand, M., C. Presant, L. Klein, Iatrogenic superior vena cava syndrome. A new entity. *Cancer* 1984; **54**: 376–378.

[21] Capek, P. and C. Cope, Percutaneous Treatment of Superior Vena Cava Syndrome. *AJR* 1989; **152**: 183–184.

[22] Furui, S., S. Sawada, K. Kuramoto, Y. Inoue, T. Irie, K. Makita, T. Yamauchi, K. Tsuchiya and S. Kusano, Gianturco-Stent placement in malignant caval obstruction: Analysis of factors predicting the outcome. *Radiology* 1995; **195**: 147–152.

[23] Dyet, J.F., A.A. Nicholson and A.M. Cook, The Use of the Wallstent Endovascular Prothesis in the Treatment of Malignant Obstruction of the Superior Vena Cava. *Clin. Radiol.* 1993; **48**: 381–385.

[24] Oudkerk, M., M. Heystraten and G. Stoter, Stenting in malignant vena caval obstruction. *Cancer* 1993; **71**: 142–146.

[25] Stanford, W. and D.B. Doty, The role of venography and surgery in the management of patients with superior vena cava obstruction. *Radiology* 1986; **160**: 845.

[26] Gooding, G.A., D.R. Hightower and E.H. Moore, Obstruction of the superior vena cava or subclavian veins: sonographic diagnosis. *Radiology* 1986; **159**: 663–665.

[27] Nieto, A.F. and D.B. Doty, Superior vena cava obstruction. Clinical syndrome, etiology, and treatment. *Curr. Probl. Cancer*. 1986; 442–484.

[28] Laissy, J.P., C. Grand, C. Matos, J. Struyven, J.F. Berger and E. Schouman-Claeys, Magnetic resonance angiography of intravascular endoprotheses: investigation of three devices. *Cardiovasc. Intervent. Radiol.* 1995; **18**: 360–366.

[29] Beck, C., W. Berberich, A. Bauknecht and K. Schnabel, Die obere Einflusstauung als Notfall in der Strahlentherapie. *Strahlenther. Onkol.* 1990; **166**: 798–802.

[30] Chen, J.C., F. Bongard and S.R. Klein, A contemporary perspective on superior vena cava syndrome. *Am. J. Surg.* 1990; **160**: 207–211.

[31] Doty, D.B., Bypass of superior vena cava: six years experience with spiral vein graft for obstruction of superior vena cava due to benign or malignant disease. *J. Thorac. Cardiovasc. Surg.* 1982; **83**: 326–338.

[32] Sculier, J.P., W.K. Evans and R. Feld, Superior vena caval obstruction syndrome in small cell lung cancer. *Cancer* 1986; **57**: 847–851.

[33] Spiro, S.G., S. Shah, P.G. Harper, J.S. Tobias, D.M. Geddes and R.L. Souhami, Treatment of obstruction of the superior vena cava by combination chemotherapy with and without irradiation in small cell carcinoma of the bronchus. *Thorax* 1983; **38**: 501–505.

[34] Jackson, J.E. and D.M. Brooks, Stenting of superior vena caval obstruction. *Thorax* 1995; 50 (Suppl 1): S31–S36.

[35] Gloviczki, P., P.C. Pairolero, K.J. Cherry and J.W. Hallett, Reconstruction of the vena cava and of its tributaries: a preliminary report. *J. Vasc. Surg.* 1990; **11**: 373–381.

[36] Carrasco, C.H., C. Charnsangavej, K.C. Wright, S. Wallace and C. Gianturco, Use of the Gianturco self-expanding stent in stenoses of the superior and inferior venae cavae. *J. of Vasc. and Intervent. Radiol.* 1992; **3**: 409–419.

[37] Charnsangavej, C., C.H. Carrasco, S. Wallace, K.C. Wright, K. Ogawa and W. Richli, Stenosis of the vena cava: preliminary assessment of treatment with expandable metallic stents. *Radiology* 1986; **161**: 295–298.

[38] Gianturco (inventor), Cook, Inc Percutaneous Endovascular Stent and Method for Insertion thereof. U.S. patent 4,580,568, 1984.

[39] Tacke, J., F. Antonucci, G. Stuckmann, R. Mattias, N. Espinosa and C.L. Zollikofer Palliative Therapie venöser Stenosen bei Tumorpatienten mit selbstexpandierenden Gefaßendoprothesen. *Rofo. Fortschr.* 1994; **160**: 433–440.

[40] Wright, K., S. Wallace, C. Charnsangavej, C. Carrasco and C. Gianturco, Percutaneous endovascular stents. An experimental evaluation. *Radiology* 1985; **156**: 69–72.

[41] Bartorelli, A.L., F. Fabiocchi, P. Montorsi, A. Loaldi, G. Tamborini and P. Sganzerla, Successful transcatheter management of Palmaz stent embolization after superior vena cava stenting. *Cathet. Cardiovasc. Diagn.* 1995; **34**: 162–166.

[42] Dodds, G.A., J.K. Harrison, M.P. O'Laughlin, J.S. Wilson, K.B. Kisslo and T.M. Bashore, Relief of superior vena cava syndrome due to fibrosing mediastinitis using the Palmaz stent. *Chest* 1994; **106**: 315–318.

[43] Elson, J.D., G.J. Becker, M.H. Wholey and K.O. Ehrmann, Vena caval and central venous stenoses: management with Palmaz balloon-expandable intraluminal stents. *J. Vasc. Interv. Radiol.* 1991; **2**: 215–223.

[44] Solomon, N., M.H. Holey and C.R. Jarmolowski, Intravascular stents in the management of superior vena cava syndrome. *Cath. Cardiovasc. Diagn.* 1991; **23**: 245–252.

[45] Edwards, R.D. and J.E. Jackson, Case Report: Superior Vena Caval Obstruction Treated by Thrombolysis, Mechanical Thrombectomy and Metallic Stents. *Clin. Radiol.* 1993; **48**: 215–217.

[46] Gray, B.H., J.W. Olin and R.A. Graor, Safety and efficacy of thrombolytic therapy for superior vena cava syndrome. *Chest* 1991; **99**: 54–59.

[47] Greenberg, S., R. Kosinski and J. Daniels, Treatment of superior vena cava thrombosis with recombinant tissue type plasminogen activator. *Chest* 1991; **99**: 1298–1301.

[48] Ferro, C., A. Scarrone, M. Borrelli, G. Buccheri, D. Ferrigno, G. Marchetti, E. Russi, F. Perona and A. Barile, Trattamento della sindrome della vena cava superiore constent metallici. Esperienza preliminare. *Radiol. Med. Torino.* 1995; **90**: 457–462.

[49] Link, J., J. Brossmann, S. Müller-Hulsbeeck and M. Heller, Venöse Stentapplikation mit simultanem kubitofemoralen Zugang. *Rofo. Fortschr.* 1995; **163**: 81–83.

[50] Sawada, S., Y. Fujiwara, T. Koyama, M. Kobayashi, N. Tanigawa, T. Iwamiya, Y. Katsube, H. Nakamura and S. Furui, Application of Expandable Metallic Stents to the Venous System. *Acta. Radiol.* 1992; **33**: 156–159.

[51] Wilhelm, K., H. Schild, J. Textor, P. Mildenberger, H. Strunk, B. Terjung and J. Lorenz, Stent-Implantation zur palliativen Therapie der oberen Einflußstauung bei Bronchialkarzinom. *DMW* 1995; **120**: 1419–1425.

[52] Antonucci, F., E. Salomonowitz, G. Stuckmann, M. Stiefel, J. Largiader and C.L. Zollikofer, Placement of venous stents: clinical experience with a self-expanding prosthesis. *Radiology* 1992; **183**: 493–497.

[53] Gaines, P.A., A.M. Belli, P.B. Anderson, K. McBride and A.P. Hemingway, Superior vena caval obstruction managed by Gianturco-Z-stent. *Clin. Radiol.* 1994; **49**: 202–206.

[54] Ohler, W., Antithrombotika und Fibrinolysetherapie. In: Weihrauch T.R. (Eds): *Internistische Therapie 1994/95*, 10th edition, Urban & Schwarzenberg, München, 1994.

[55] Crowe, T., C.H. Davies and P.A. Gaines, Percutaneous management of superior vena cava occlusions. *Cardiovasc. Intervent. Radiol.* 1995; **18**: 367–372.

[56] Rosch, J., J.E. Bedell, J. Putnam, R. Antonovic and B. Uchida, Gianturco expandable wire stents in the treatment of superior vena cava syndrome recurring after maximum-tolerance radiation. *Cancer* 1987; **60**: 1243–1246.

[57] Spiro, S.G. In: RAL Brewis, G.J. Gibson and D.M. Geddes (Eds.): *Respiratory Medicine*. Tindall, London 1990, pp. 867–868.

